# Enterotoxigenic *Escherichia coli* as a Modulator of the Entero-Pulmonary Axis in Piglets: Impacts on the Microbiota and Immune Responses

**DOI:** 10.1155/tbed/8865503

**Published:** 2025-02-22

**Authors:** Gabriela Merker Breyer, Silvia De Carli, Maria Eduarda Rocha Jacques da Silva, Maria Eduarda Dias, Ana Paula Muterle Varela, Michele Bertoni Mann, Jeverson Frazzon, Fabiana Quoos Mayer, Itabajara da Silva Vaz Junior, Franciele Maboni Siqueira

**Affiliations:** ^1^Laboratory of Veterinary Bacteriology, Department of Veterinary Pathology, Federal University of Rio Grande do Sul, Porto Alegre, Rio Grande Do Sul, Brazil; ^2^Postgraduate Program in Veterinary Sciences, Federal University of Rio Grande do Sul, Porto Alegre, Rio Grande Do Sul, Brazil; ^3^Postgraduate Program in Biosciences, Federal University of Health Sciences of Porto Alegre, Porto Alegre, Rio Grande Do Sul, Brazil; ^4^Postgraduate Program in Agricultural and Environmental Microbiology, Federal University of Rio Grande do Sul, Porto Alegre, Rio Grande Do Sul, Brazil; ^5^Laboratory of Biochemistry and Molecular Biology of Microorganisms, Department of Food Science, Federal University of Rio Grande do Sul, Porto Alegre, Rio Grande Do Sul, Brazil; ^6^Animal Health Research Center, Department of Agricultural Diagnosis and Research, Desidério Finamor Veterinary Research Institute, Eldorado do Sul, Rio Grande Do Sul, Brazil; ^7^Department of Molecular Biology and Biotechnology, Federal University of Rio Grande do Sul, Porto Alegre, Rio Grande Do Sul, Brazil; ^8^Biotechnology Center, Federal University of Rio Grande do Sul, Porto Alegre, Rio Grande Do Sul, Brazil

**Keywords:** 16S-rDNA sequencing, biomarker bacteria, ETEC, immune markers, postweaning diarrhea

## Abstract

The high prevalence of enterotoxigenic *Escherichia coli* (ETEC) in nondiarrheic piglets contributes to its rapid spread; however, few studies have explored the effects of latent gastrointestinal pathogens on animal health. Therefore, using high-throughput sequencing approaches, we explored changes in entero-pulmonary microbiota and immune gene expression in healthy, asymptomatic, and diarrheic piglets. As expected, bacterial communities were less diverse in the respiratory tract than in the gut, with a site-specific composition that was more stable in the gut and highly variable in the lung among the investigated animals. Although no significant changes in diversity rates were seen based on ETEC-carrier state, our findings suggest that ETEC's presence can cause dysbiosis in the gut and lung in asymptomatic and diarrheic piglets, reinforcing the crosstalk in the entero-pulmonary axis. We also identified potential bacterial biomarkers that can be used to monitor piglet health: *Sphaerochaeta*, *Bacteroides*, *Butyricoccus*, and *Blautia* were highly represented in the gut, while *Streptococcus* and *Prevotellaceae* NK3B31 group were enriched in the lungs of healthy piglets. In addition, most metabolic pathways predicted in the bacterial communities were shared despite the ETEC-carrier state, with differences observed only in the gut microbiota, suggesting that ETEC's presence may impact substrate utilization. Finally, we observed shifts in the intestinal expression of *tff2* and *cd36* immune markers between healthy and diarrheic piglets, which might suggest their use as prognostic markers for postweaning diarrhea (PWD). Although the effect remains unclear, the ETEC-carrier state also altered the transcription of other markers locally (in the gut and lung) and systemically, which corroborates the shared mucosal immunity in the entero-pulmonary axis in piglets. Overall, despite limitations regarding sample size, our findings give clues about the entero-pulmonary dynamics in piglets in the presence of a gastrointestinal pathogen, representing a starting point for future research on this axis for veterinary purposes.

## 1. Introduction

Enterotoxigenic *Escherichia coli* (ETEC) causes postweaning diarrhea (PWD) in weaned piglets, leading to a mortality rate of up to 25% in infected litters [1]. To succeed, the infection requires not only the ingestion of the pathogen but also the expression of specific receptors in the host's gastrointestinal tract for bacterial colonization and enterotoxin activity [2]. Thus, even nondiarrheic pigs can act as a reservoir and contribute to ETEC's dissemination [3], showing a prevalence of up to 66% in asymptomatic piglets at nursery and causing recurrent PWD outbreaks in the same herds [4]. A previous study using an ETEC-induced infection model in pigs determined that ETEC-related diarrhea shifts the gut bacterial community, showing a lower Bacteroidota:Firmicutes ratio with a lower abundance of *Prevotella* and a higher abundance of *Lactococcus* and *Escherichia-Shigella* in comparison to healthy animals [5]. However, even though gut microbiota dysbiosis has been reported during induced-ETEC infections, the implications of the ETEC-carrier state on pig health in the swine farms environment, with natural exposure to the pathogen, remains uncertain.

Furthermore, the presence of gastrointestinal pathogens in mammalian hosts modulates not only the intestinal microbiota but also other sites, impacting several physiological functions, including metabolic homeostasis, immune response/stimulation, and epithelial maturation [6–8]. Due to the interaction between mucosal membranes, changes in the intestinal microbiota can be extended to the respiratory tract [9]. Several studies have recently investigated the entero-pulmonary axis in humans [10, 11] or mouse models [7, 12], but only a few have investigated pigs for veterinary purposes [13]. Previous studies in humans have determined that high abundances of intestinal *E. coli* are associated with increased occurrence of bacterial and viral respiratory diseases [14–16], enhancing the risk of opportunist infections. However, the impacts of ETEC on host respiratory health are still unexplored to date. Thus, we investigated the impacts of the ETEC-carrier state, considering both asymptomatic and diarrheic piglets, on mucosal health, including changes in the entero-pulmonary microbiota and the immune gene expression, with the aim of better understanding the dynamics of the entero-pulmonary axis in pig farms with natural ETEC exposure.

## 2. Material and Methods

### 2.1. Animals and Sample Collection

This study underwent ethical review and was approved by an Institutional Animal Care and Use Committee under number CEUA/UFRGS–33558. All methods were carried out in accordance with Animal Welfare Guidelines and Policies.

The sampling was conducted by convenience. A total of 30 nursery piglets (28–42 days) from the same litter were analyzed, and their relevant clinical history was assessed. All animals had no history of antibiotic therapy in the 30 days prior to the collection.

Stool samples were collected directly from the rectum into sterile microtubes; blood was collected in 3.2% sodium citrate and 3.5 mL blood collection tubes (Greiner Bio-One, São Paulo, Brazil); and cecum and lung tissue were collected immediately after slaughter. All samples were stored at 4°C until sample processing, which occurred within 12 h.

### 2.2. Nucleic Acids Isolation

Metagenomic DNA of stool and lung samples was isolated using the DNeasy PowerSoil kit (Qiagen, Hilden, Germany) according to the manufacturer's instructions. For lung samples, 40 mg of tissue was subjected to a pre-lysis treatment with 1 mg lysozyme (Sigma–Aldrich, Missouri, USA) at 37 °C for 90 min and 0.2 mg proteinase K (Thermo Fisher Scientific, Massachusetts, USA) at 65 °C for 90 min. DNA quality and quantification were determined using a NanoDrop 2000 (Thermo Fisher Scientific, Massachusetts, USA) and a Qubit 2.0 fluorometer with the Qubit dsDNA BR assay kit (Thermo Fisher Scientific, Massachusetts, USA).

Total RNA from blood, cecum, and lung samples was isolated using TRIzol (Invitrogen, Massachusetts, USA). In detail, white blood cells (WBCs) were recovered from blood samples by centrifugation (3500× *g*; 5 min); for cecum and lung samples, 20 mg were macerated with micropistils and liquid nitrogen. Then, 500 μL TRIzol was added to each sample, and RNA isolation followed manufacturers' instructions. RNA integrity was analyzed by 1% agarose gel electrophoresis; then, quality and quantification were assessed by NanoDrop 2000 (Thermo Fisher Scientific, Massachusetts, USA). Only RNA samples with high integrity and quality (A_260:280_ = 1.8–2.2) were eligible for further analyses.

### 2.3. ETEC Marker Screening

We performed a molecular screening targeting five porcine ETEC-markers, including enterotoxins Sta (*estA*), STb (*estB*), and LT (*elt*), and both fimbrial adhesins F4 (*fae*) and F5 (*fan*) [17, 18]. PCR reactions were performed in 25 µL containing 1x buffer, 0.2 mM dNTP, 2.0 mM MgSO_4_, 0.2 µM of each primer, 1 U Taq DNA Polymerase Recombinant (Invitrogen, Massachusetts, USA), and 50 ng of fecal DNA. Cycling conditions included an initial at 94°C for 5 min, 30 cycles of 45 s at 94°C, 30 s at 50°C, and 30 s at 72°C and a final extension at 72°C for 5 min. PCR products were evaluated by electrophoresis in 2% agarose gel stained with 40x UniSafe Dye (Uniscience Corporation, Florida, USA). Piglets were classified as noncarriers (ETEC−) when no ETEC-marker was detected and as ETEC-carriers (ETEC+) when at least one of the investigated enterotoxins (LT, STa, and/or STb) was detected, as the development of PWD depends of enterotoxin production and activity.

### 2.4. Classification of the Piglets Based on ETEC Presence and Clinical Signs

We selected 20 piglets for further analyses based on ETEC-carrier state (ETEC+ or ETEC−) and the occurrence of gastrointestinal clinical signs in the 3 days prior to collection. Animals were grouped as follows: group A: healthy ETEC− piglets (*n* = 3); group B: asymptomatic ETEC+ piglets (*n* = 10); and group C: diarrheic ETEC+ piglets (*n* = 7).

### 2.5. S-rDNA Sequencing and Taxonomic Assignment

We targeted 16 S-rDNA (V4 region) using the universal 515F and 806R primers [19] with overhang Illumina adapters in 50 µL amplicon-PCR reactions, containing 1x buffer, 0.2 mM dNTP, 2.0 mM MgSO_4_, 0.5 µM of each primer, and 1 U Platinum Taq DNA Polymerase High Fidelity (Invitrogen, Massachusetts, USA), using 12.5 ng stool DNA and 500 ng lung DNA as template [20]. Cycling conditions were: 94°C for 3 min, followed by 25 cycles of 94°C for 30 s, 55°C for 30 s, and 72°C for 30 s, and finally 72°C for 3 min. PCR amplicons were assessed by 1% agarose gel electrophoresis with 40x UniSafe Dye (Uniscience Corporation, Florida, USA), then subjected to pair-end 2 × 250 bp sequencing with Illumina MiSeq (Illumina, California, USA) following the manufacturer's instructions (MiSeq Reagent Kit v2, 500 cycle).

Raw reads were evaluated using FastQC (v0.11.9) [21]; then, low-quality sequences, short-length reads (<80 nt with HEADCROP = 20), and primer and adaptor sequences were removed by Trimmomatic [22]. QIIIME2 v2020.2 [23] with the DADA2 package [24] was used to merge paired-end sequences, remove chimeras, and cluster reads into amplicon sequence variants (ASVs) with truncation length of 150–130. ASV taxonomic assignment was performed with the Silva 138.1 database (classifier silva-138-99-515-806-nb-classifier.qza, 2022 version) [25]. Eukaryote, Archaea, and unknown sequences were removed from the sample libraries for further analyses.

### 2.6. Metataxonomic Analyses

Bacterial communities were analyzed by the phyloseq package [26] in RStudio. Entero-pulmonary axis diversity was analyzed by comparing the investigated groups (A, B, and C). Chao1 and Shannon *α*-diversity indices were estimated by the Kruskal–Wallis test with Wilcoxon pairwise comparison (*p* < 0.05). Permutational multivariate analyses of variance (PERMANOVA) with 999 permutations (*p* < 0.05) compared the bacterial composition between sites and groups using vegan [27] and principal coordinates analysis (PCoA) using unweighted Unifrac metrics was performed using the microbiome package [28].

Dysbiosis in gut and lung sites was assessed using the dysbiosisR package based on Euclidean distance to group centroids with Bray–Curtis distance metrics [29]. The chosen dysbiosis score model was based on the area under the multiclass receiver operating characteristics (ROC) curve (AUC ≥ 0.85). Dysbiosis and normobiosis thresholds were calculated based on the 90^th^ percentile of the dysbiosis score in group A (healthy ETEC− piglets).

Shared taxa were investigated using the eulerr package [30] and used to build a Venn diagram comparing groups. Taxa's relative abundance in both sites was visualized using ggplot2 [31] and microbiome [28]. For each site, we also determined taxa markers for healthy, asymptomatic ETEC+, and diarrheic ETEC+ piglets using discriminant analysis effect size (LEfSe; bootstrap = 1000, *p* < 0.05) method [32] with counts per million (CPM) normalization by the microbiome Marker package [33]. Finally, we investigated the presence of relevant classical respiratory bacterial pathogens for swine in lung microbiota, including *Mycoplasma*, *Actinobacillus*, *Bordetella*, *Pasteurella*, and *Glasserella/Haemophilus* [34].

### 2.7. Bacterial Functional Prediction of the Piglets' Entero-Pulmonary Axis

Functional prediction of piglets' entero-pulmonary axis based on 16S-rDNA was performed using PICRUSt2 [35] using the MetaCyc Metabolic Pathway Database [36]. Statistical analysis and visualization were performed by the ggpicrust2 package [37] in RStudio. Principal component analysis (PCA) was performed to assess the distribution of the predicted pathways in the investigated groups and linear models for differential abundance analysis of microbiome compositional data (LinDA; *p* < 0.05) [38] were performed using healthy piglets as reference.

### 2.8. Transcriptional Profile of Piglets' Immune Markers

Reverse transcription reactions using the Moloney Murine Leukemia Virus Reverse Transcriptase kit (MMLV-RT; Invitrogen, Massachusetts, USA) and Oligo (dT)12–18 primers (Invitrogen, Massachusetts, USA) with 500 ng RNA were performed following the manufacturer's instructions. Quantitative PCR was performed in 10 µL reactions using the PowerUp SYBR Green Master mix (Applied Biosystem, Massachusetts, USA) with 10 ng cDNA in an Applied Biosystems 7500 Fast Real-Time PCR System (Thermo Fisher Scientific, Massachusetts, USA). A total of 11 immune marker genes were investigated in the gut, lung, and WBCs, including targets coding for interleukins IL1*β*, IL4, IL6, IL10, and IL22; mucin MUC2; trefoil factor TFF2; platelet glycoprotein CD36; antibacterial peptide PMAP36; defensin DEF1; and tumor necrosis factor (TNF)-*α* (Supporting Information 1: Table S1). *β-actin* was used for normalization. Melting curves were performed to check primers specificity. Each biological sample was tested in three technical replicates.

Relative gene expression was calculated by the PFFAFL method [39], considering primers' efficiency according to RDML-LinRegPCR [40] (Supporting Information 1: Table S1). Statistical analysis comparing relative mRNA expression (log2 fold change) among groups was performed by one-way analyses of variance (ANOVA) with Tukey's multiple comparisons test (*p* < 0.05) and the Kruskal–Wallis test with Dunn's multiple comparisons test (*p* < 0.05) for parametric and nonparametric data, respectively, using GraphPad Prism v.7 software.

## 3. Results

### 3.1. ETEC-Carrier State Impact on Entero-Pulmonary Dysbiosis

The investigation of the ETEC-carrier state by molecular and clinical analyses identified only three healthy ETEC− piglets (10%) with no clinical sign of infection; 90% harbored at least one ETEC marker, with 16 asymptomatic and 11 diarrheic piglets. From these, 20 animals were selected for further analyses.

**Figure 1:**
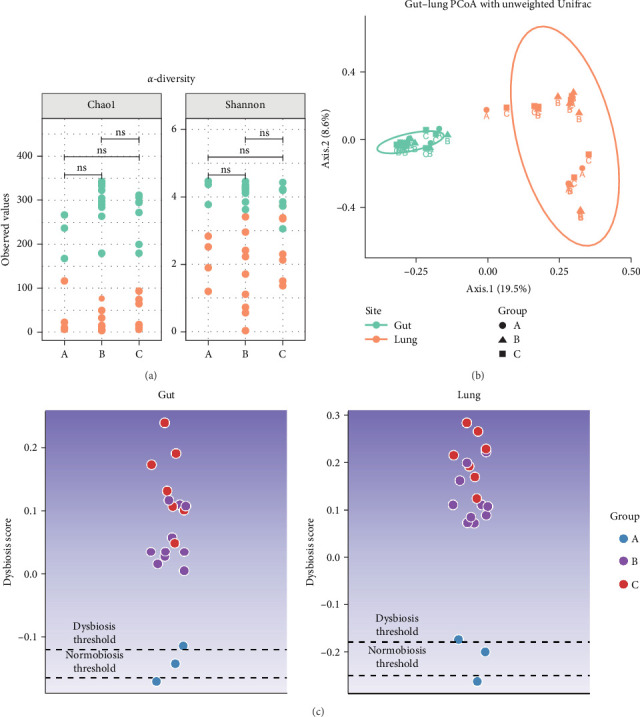
Microbiota diversity in the entero-pulmonary axis in healthy (Group A), asymptomatic ETEC+ (Group B), and diarrheic ETEC+ piglets (Group C). (A) Chao1 and Shannon *α*-diversity indices (*p* < 0.05). (B) Principal coordinates analysis (PCoA) with unweighted Unifrac metrics (*p* < 0.05). (C) Entero-pulmonary dysbiosis score. Dysbiosis and normobiosis thresholds were calculated based on the 90^th^ percentile of the dysbiosis score (AUC_gut_ = 0.9524 and AUC_lung_ = 0.9619). ETEC, enterotoxigenic *Escherichia coli*.

Microbiota investigation showed that both gut and lung bacterial communities presented similar diversity rates despite the ETEC-carrier state (*p* > 0.05; data not shown). However, comparing entero-pulmonary axis, the lung microbiota was less diverse than the gut microbiota (*p* < 0.05; Figure 1A), significantly impacting on the bacterial composition based on PERMANOVA (*p*=0.001; R^2^ = 0.15558; Figure 1B). Nevertheless, dysbiosis score analysis indicated dysbiosis in all asymptomatic and diarrheic ETEC+ piglets (groups B and C) based on the healthy ETEC− dysbiosis score (Figure 1C).

### 3.2. Changes in Entero-Pulmonary Bacterial Communities are Observed in ETEC-Carrier Piglets

**Figure 2:**
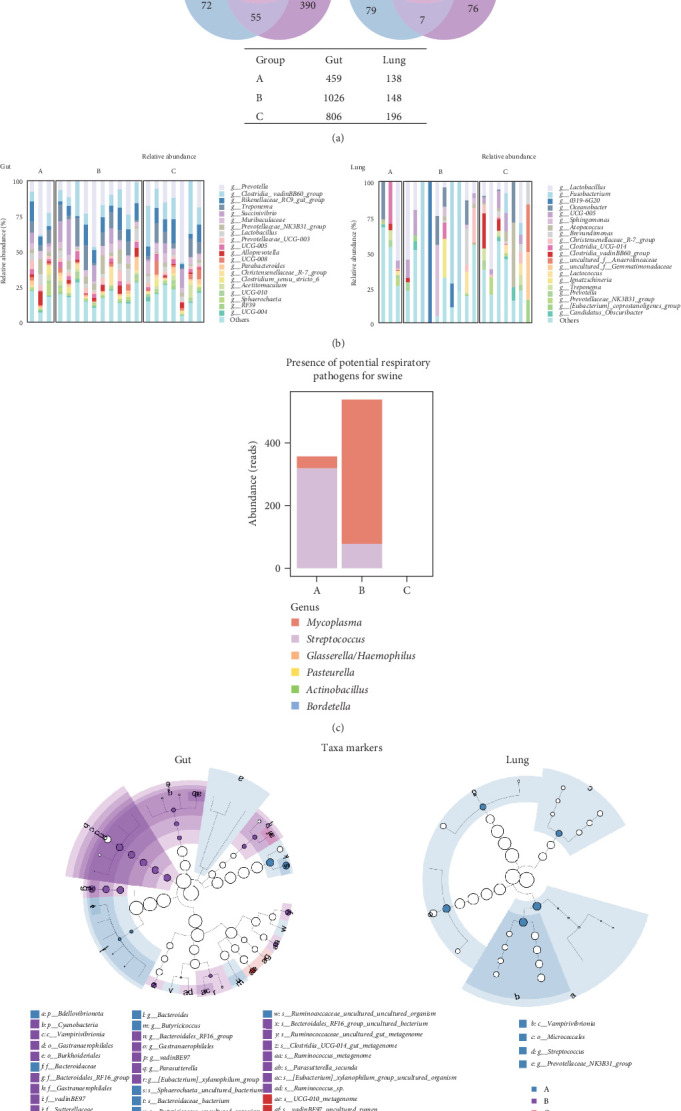
Bacterial composition of healthy (Group A), asymptomatic ETEC+ (Group B), and diarrheic ETEC+ piglets (Group C) in the entero-pulmonary axis. (A) Venn diagram based on the shared core taxa among groups. (B) Genus relative abundance. (C) Search of classical pathogenic bacteria that are potentially involved in pig's respiratory infections. (D) Taxa markers for each group based on discriminant analysis effect size (LEfSe; bootstrap = 1000, *p*  < 0.05) method with CPM normalization. CPM, counts per million; ETEC, enterotoxigenic *Escherichia coli*.

To assess whether the ETEC-carrier state modulates entero-pulmonary microbiota, we compared the bacterial composition in each site. We identified 315 shared taxa among the analyzed groups in the gut microbiota, whereas only 37 core taxa were shared in the lung microbiota; noteworthily, ETEC− piglets (group A) presented fewer taxa than piglets in the ETEC+ groups (B and C; Figure 2A). Furthermore, we observed stability in the relative abundance of the most abundant genera in the gut microbiota, which was enriched in *Prevotella*, the *Clostridia* vadin BB60 group, the *Rikenellaceae* RC9 gut group, and *Treponema*; in contrast, the lung microbiota's bacterial composition was highly variable among the analyzed animals and groups (Figure 2B).

To evaluate ETEC's impact on piglets' respiratory health, we searched for potentially pathogenic bacteria in the lung microbiota. We only identified *Mycoplasma* and *Streptococcus* in healthy ETEC− and asymptomatic ETEC+ piglets (Figure 2C). *Actinobacillus*, *Bordetella*, *Pasteurella*, and *Glasserella/Haemophilus* were not detected in the analyzed samples. Additionally, biomarker analysis identified 33 taxa in piglets' guts and five markers in their lungs based on ETEC-carrier state (Figure 2D). Considering only genus- and species-level markers, healthy piglets were marked by *Sphaerochaeta*, *Bacteroides*, *Butyricicoccus*, and *Blautia* in the gut, and by *Streptococcus* and the *Prevotellaceae* NK3B31 group in the lungs (Table 1). Asymptomatic ETEC+ piglets' gut markers included *Ruminococcus*, *Parasutterella*/*P. secunda*, and the [*Eubacterium*] *xylanophilum* group; and diarrheic ETEC+ piglets' guts were marked by the [*Eubacterium*] *coprostanoligenes* group (Table 1).

### 3.3. Differences in Metabolic Pathways Associated to ETEC-Carrier State are Limited to Piglets' Gut

**Figure 3:**
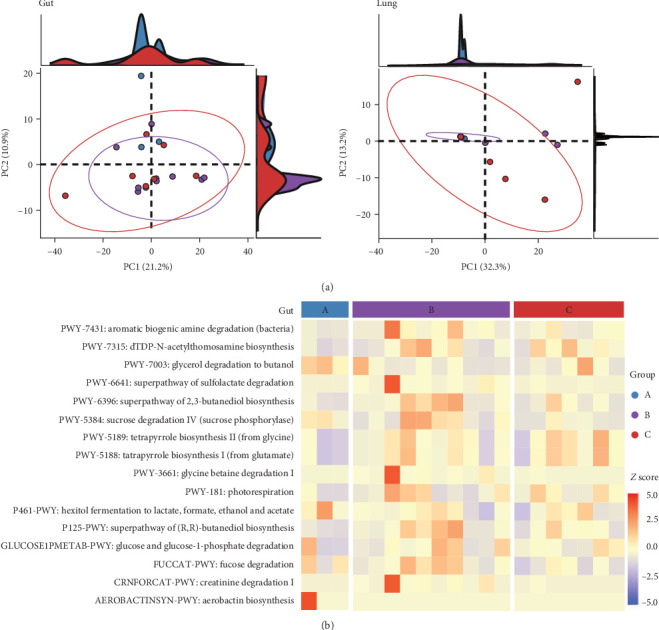
PICRUSt2 functional prediction based on the bacterial communities in pigs' entero-pulmonary axis, considering healthy (Group A; blue), asymptomatic ETEC+ (Group B; purple), and diarrheic ETEC+ piglets (Group C; red). (A) Principal component analysis (PCA) distribution of metabolic pathways. (B) Heatmap of functional pathways with differential abundance in gut (LinDA; *p* < 0.05) based on the MetaCyc database. ETEC, enterotoxigenic *Escherichia coli*.

To assess the functional implications of ETEC-carrier state in piglets' entero-pulmonary axis, we predicted and compared the metabolic pathways that potentially harbored the bacterial communities in the investigated groups. In both sites, PCA showed similar metabolic pathway distributions among healthy, asymptomatic ETEC+, and diarrheic ETEC+ piglets (Figure 3A). Significant differential abundance was observed only in the gut, where 16 pathways related to biosynthesis, substrate degradation/utilization, and fermentation were identified in comparison to healthy ETEC− piglets (*p* < 0.05; Figure 3B; Supporting Information 2: Table S2).

### 3.4. The Expression of Immune Markers in Piglets' Entero-Pulmonary Axis and Systemically are Affected by the ETEC-Carrier State

**Figure 4:**
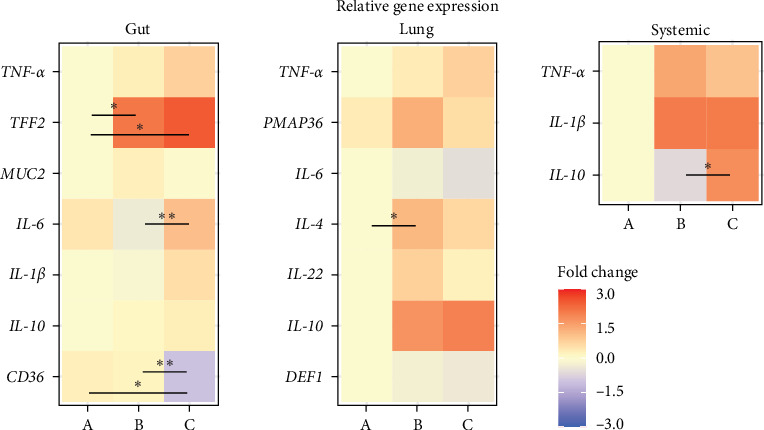
RT-qPCR relative expression of immune markers in piglets. Heatmap of local and systemic mRNA fold change in healthy (group A), asymptomatic ETEC+ (group B), and diarrheic ETEC+ (group C) piglets. Statistical differences among groups based on one-way analyses of variance (ANOVA) with Tukey's multiple comparisons test or Kruskal–Wallis with Dunn's multiple comparison test (*p* < 0.05) for parametric and nonparametric data, respectively, are indicated when *p* < 0.05 (*⁣*^*∗*^) or *p*  < 0.01(*⁣*^*∗∗*^). ETEC, enterotoxigenic *Escherichia coli*.

Finally, the role of ETEC-carrier state in the expression of piglets' immune markers was determined by comparing relative gene expression among the groups in each site. Intestinal expression of *tff2* was upregulated in asymptomatic (group B) and diarrheic ETEC+ (group C), and *cd36* was downregulated in diarrheic ETEC+ piglets (group C; *p* < 0.05) when compared to healthy animals (group A). We also observed significant differences in the *il6* and *cd36* genes between piglets from group B and group C (*p* < 0.05; Figure 4). Interestingly, we also observed changes in the expression levels of immune markers in the lung and systemically (Figure 4): in the lung, *il4* was upregulated in asymptomatic ETEC+ (group B) compared to healthy piglets (group A; *p* < 0.05); whereas systemically, *il10* was upregulated in diarrheic ETEC+ piglets (group C) in comparison to asymptomatic ETEC+ piglets (group B; *p* < 0.05). Although without statistical significance in gut and lung tissues, our findings suggest differential expression of *il10* in the ETEC-carrier piglets (groups B and C) compared to healthy ETEC− piglets (group A), as shown in Figure 4.

## 4. Discussion

The high prevalence of ETEC on swine farms contributes to the pathogen's quick spread within contaminated litters, leading to significant economic losses for producers [3, 4, 41, 42]. The effects of ETEC-carrier state on the health of asymptomatic pigs are rarely explored in veterinary studies [18], which often focus on studying pigs with clinical signs or using ETEC-induced models [5, 43, 44]; hence, this study aimed to determine whether ETEC modulates the entero-pulmonary microbiota and the immune response in nursery piglets considering a pig farm environment with natural exposure to ETEC.

The molecular detection of ETEC markers indicated a high occurrence of ETEC in the investigated piglets (90%), which corroborates the high prevalence rates in the pig litters [4]. This observation made it difficult to gather piglets for the control group, limiting its sample size and consequently leading to a more delicate interpretation of the data; however, given the unprecedentedness of such analysis, our findings still give a clue about ETEC's role in the entero-pulmonary axis in piglets in situ. Notably, the gut microbiota continuously interacts with and influences intestinal stem cells (ISCs), which are responsible for the renewal of the intestinal epithelium and its homeostasis [8]. The presence of ETEC, specifically due to the acute production of heat-stable enterotoxins, causes epithelial injuries that damage cell barriers' integrity and is associated with the occurrence and development of diarrhea [45] Moreover, given that the gastrointestinal and respiratory tracts are part of a shared mucosal immune system, the presence of a pathogen in one organ may modulate the microbiota and immune response in the entero-pulmonary axis [46]. Therefore, despite ETEC's high occurrence in the litter (90%), diarrhea was only observed in 41% of the ETEC+ piglets (11/27); perhaps disease development might occur due to the ISC niche dysregulation triggered by the animals' gut microbiota.

In this study, bacterial communities were less diverse in the respiratory tract than in the gut with a site-specific composition, as previously observed in humans and pigs [47, 48]. *Prevotella*, the *Rikenellaceae* RC9 gut group, the *Clostridia* vadin BB60 group, and *Treponema* were highly abundant in the gut microbiota in general. *Prevotella* is part of piglets' commensal gut microbiota [49] and has been reported in both healthy and diarrheic piglets [50, 51]; the *Rikenellaceae* RC9 gut group showed a positive association with weight gain during the weaning stage [52]; meanwhile, the *Clostridia* vadin BB60 group and *Treponema* are correlated with better feed efficiency, being commonly found along the pig's gastrointestinal tract [50, 51, 53, 54]. In contrast, the lung microbiota was highly variable in this study, with low bacterial diversity, which made robust interpretation difficult. Nevertheless, most piglets presented *Lactobacillus* in their lungs, which is reported as one of the most abundant genera in the upper respiratory tract of healthy pigs [55].

To understand how the ETEC-carrier state would modulate the microbiota of the entero-pulmonary axis in nursery piglets, we compared the bacterial communities in healthy, asymptomatic ETEC+, and diarrheic animals. No change was observed in the diversity rates due to the ETEC-carrier state; however, despite the limited sample size in the healthy ETEC− group, the dysbiosis analysis suggested an association between ETEC's presence and alterations in both gut and lung bacterial communities, since asymptomatic and diarrheic ETEC+ piglets showed elevated dysbiosis scores compared to healthy ETEC− piglets. Such observation corroborates a previous study using an ETEC-induced model that also reported dysbiosis in gut microbiota due to ETEC infection [5].

We identified exclusive taxa in the gut and lung for all ETEC-carrier states and potential bacterial markers that can be used to monitor piglets' health. Interestingly, healthy ETEC− piglets were the only group with taxa markers in both sites (gut and lung). In detail, the gut microbiota of healthy animals was marked by *Bacteroides*, which is considered a component of core microbiota in this site [53]; *Sphaerochaeta* and *Butyricicoccus*, which have already been described in pigs' gut, are related to greater energy harvest [56] and better feed conversion [57], respectively. In lung microbiota, healthy ETEC− piglets were marked by *Streptococcus*, which is a major genus found in the porcine upper respiratory tract in healthy individuals being mostly related to commensal species [55], suggesting that ETEC's presence might impact *Streptococcus* balance in the respiratory tract.

Asymptomatic ETEC+ piglets' gut microbiota was marked by *Parasutterella*, which contributes to inflammation during diarrhea [58], and *Ruminococcus*, a ubiquitous bacterium commonly found in pigs' fecal microbiota that is reported to be less abundant in diarrheic piglets [58, 59]. Furthermore, [*Eubacterium*] *coprostanoligenes* was the only biomarker in diarrheic ETEC+ piglets, which increases its abundance when piglets are under oxidative stress [60]. The lack of biomarkers in the diarrheic group points to the potential bacterial imbalance in the entero-pulmonary axis during ETEC infection, corroborating the results of the dysbiosis analysis.

Additionally, to assess whether the ETEC-carrier state influenced the lung microbiota regarding classical pathogenic bacteria related to respiratory diseases in swine, we compared the abundance rates of *Mycoplasma*, *Actinobacillus*, *Bordetella*, *Pasteurella*, *Glasserella*, and *Streptococcus* in the investigated groups. Interestingly, only *Mycoplasma* and *Streptococcus* were detected, in both healthy ETEC− and asymptomatic ETEC+ piglets, supporting the previous description of these bacterial genera as belonging to the commensal lung microbiota [48]. These two bacterial genera's absence in diarrheic ETEC+ piglets also corroborates a dysbiosis scenario, in which PWD might interfere in the respiratory tract modulating its bacterial community.

The analysis of functional metabolic pathways showed that the ETEC-carrier state has a low impact on the metabolic routes harboring the bacterial communities of the entero-pulmonary axis in piglets, being limited to the intestinal tract. Moreover, changes in functional pathways in the gut in the asymptomatic and diarrheic ETEC+ groups suggest that ETEC's presence might impact substrate utilization. Nevertheless, such results must be interpreted carefully due to limitations in predicting metabolic pathways using 16S-rDNA data, such as gene annotation inaccuracies in current databases and the inequivalence of taxonomic affiliation and metabolic pathways [61].

RT-qPCR analysis showed changes in the relative expression of immune markers in asymptomatic and diarrheic ETEC+ piglets, suggesting that ETEC's presence might alter the immune response at the transcriptional level in the gut and lung and systemically, even when gastrointestinal clinical signs are not reported. In the gut, ETEC's presence increased the expression of *tff2*, which encodes TFF2, which stabilizes and regenerates gastric mucus barriers and inhibits acid secretion in mammals [62–64], and the overexpression of which is associated with intestinal and respiratory inflammatory processes [65–67]. Thus, the increase in *tff2* expression levels observed in asymptomatic and diarrheic ETEC+ piglets may be a strategy to repair possible damage to the mucosal barrier caused by ETEC. Additionally, *cd36* was downregulated in diarrheic ETEC+ piglets, which encodes membrane fatty acid translocase CD36, which is involved in maintaining the intestinal barrier [68]. Fatty acids have a protective role in piglets' gut by inhibiting mucosa inflammation [69] and reducing diarrhea rates [70]. Remarkably, CD36 expression is modulated by diet and gut microbiota [71, 72] and its absence increases the susceptibility to inflammation [68]. Thus, the downregulation of *cd36* in ETEC+ diarrheic piglets is a signaling pathway for PWD. Although IL6 is a marker for intestinal inflammations in several mammals including pigs [73–75] and its increase has been reported in ETEC-induced mice using enzyme-linked immunosorbent assays [51, 53] and using RT-qPCR in pigs infected with pathogenic *E. coli* [76], our findings indicate no significant differences of *il6* expression between healthy and diarrheic piglets.

Alterations in immune genes expression due to the ETEC-carrier state were also observed in other sites by *il4* and *il10* regulation in the lung and systemically, respectively. IL4 is commonly associated with a protective effect in the gastrointestinal and respiratory tracts. A study with *Citrobacter rodentium*, a murine model for pathogenic *E. coli* infection, showed the beneficial effect of IL4 treatments for mucosal healing by affecting mucus production, pathogen adhesion, and colitis [77]. Similarly, in the respiratory tract, swine IL4 prevents porcine reproductive and respiratory syndrome (PRRS) by enhancing the immune protective response and vaccine efficacy [78]. Thus, our findings suggest that ETEC's presence in the gut might induce *il4* transcription in the lung as a defense strategy to prevent potential opportunistic infections. Finally, *il10* upregulation in WBCs was observed in diarrheic ETEC+ piglets in comparison to asymptomatic ones, indicating the systemic overexpression of this marker. Although our findings have not shown changes in *il10* relative expression in comparison to healthy ETEC− piglets, a previous study in humans demonstrated that individuals genetically predisposed to produce high levels of IL10 are more susceptible to ETEC traveler's diarrhea [79].

Overall, the gut microbiota has direct and indirect effects on entero-pulmonary crosstalk and its dysbiosis can alter immune and metabolic homeostasis, contributing to lung disease [46]. The intestinal bacterial community influences the entero-pulmonary immune system via local or long-reaching interactions by regulating T-cell populations, systemic inflammation pathways, and the production of short-chain fatty acids (SCFAs) [80]. Microbial components and metabolites can be transported systemically between the gut and lung, shaping local immune responses [81]. For instance, peptidoglycans or lipopolysaccharides are microbe-associated molecular patterns that can be recognized by host cells expressing pattern-recognition receptors, such as Toll-like receptors and NOD-like receptors. Moreover, unmetabolized SCFAs can enter host peripheral circulation and distal body sites and influence immune cell development [82]. Besides, previous studies have pointed out that the human entero-pulmonary axis exchanges immune cells directly and through host-derived mediators via circulation [83–85].

Our findings, albeit limited in terms of sample size, indicate that ETEC's presence impacts the bacterial communities in the entero-pulmonary axis, with differential expression of immune response markers in asymptomatic and diarrheic animals. Thus, implementing biosecurity measures on swine farms, such as cleaning and disinfecting the facilities between batches and following an ETEC vaccination schedule for the sows, might prevent ETEC's entry and spread in herds [86]. Moreover, ensuring a healthy gut microbiota in piglets is critical to minimize the risk of gastrointestinal pathogens like ETEC; thus, management measures must be considered, such as colostrum intake, an adequate suckling period, promoting sows' nest-building behavior, and reducing the use of antibiotics [87]. In addition, probiotic supplementation in pigs' diet might be considered, as it promotes gut microbiota homeostasis and immunity and improves the intestinal mucosal barrier, which reduces animals' susceptibility to gastrointestinal pathogens [88].

## 5. Conclusion

Considering the interactive dynamics of the entero-pulmonary axis, we investigated the ETEC-carrier state's role in piglets' microbiota and immune responses, confirming that gastrointestinal pathogens might act as modulators not only in the gut but also in the respiratory tract. We highlight that our observations need to be interpreted carefully due to the limited sample size; nevertheless, our findings are a starting point for further research about the entero-pulmonary axis in production animals.

## Figures and Tables

**Table 1:** Taxa markers for ETEC carrier state in piglets' entero-pulmonary axis.

Marker	Taxa	Enriched group	LDA score	*p*-Value
Gut				

1	*Sphaerochaeta*	A	4.228	0.027
2	*Bacteroides*	A	3.311	0.017
3	*Butyricicoccus*	A	2.675	0.015
4	*Blautia*	A	2.219	0.023
5	*Bacteroidales* RF16 group	B	3.882	0.012
6	*Clostridia* UCG-014	B	3.428	0.041
7	*Ruminococcus*	B	3.220	0.042
8	*Parasutterella*	B	3.073	0.036
9	*[Eubacterium] xylanophilum* group	B	3.055	0.048
10	*Parasutterella secunda*	B	2.981	0.032
11	*Ruminococcaceae* UCG-010	C	3.290	0.042
12	*[Eubacterium] coprostanoligenes* group	C	2.834	0.028

Lung				

1	*Streptococcus*	A	4.444	0.026
2	*Prevotellaceae* NK3B31 group	A	4.352	0.042

*Note:* A: healthy ETEC− piglets; B: asymptomatic ETEC+ piglets; and C: diarrheic ETEC+ piglets. Linear discriminant analysis effect size (LEfSe; LDA score > 2; *p* < 0.05) method [32] with CPM normalization considering genus and species levels.

Abbreviations: CPM, counts per million; ETEC, enterotoxigenic *Escherichia coli*.

## Data Availability

The data that support the findings of this study are openly available in the National Center of Biotechnology Information (NCBI) database under BioProject PRJNA1064844 with Accession numbers from SAMN39440656 to SAMN39440695.

## Supporting Information

Supporting Information 1 Table S1: Description of primer sequences used for swine immune genes transcriptional analysis by RT-qPCR.

Supporting Information 2 Table S2: Functional pathways with differential abundance in ETEC-carrier pigs in entero-pulmonary axis based on PICRUSt2 analysis.
